# The engagement of autophagy in maniac disease

**DOI:** 10.1111/cns.14353

**Published:** 2023-07-12

**Authors:** Yidong Yang, Renxiang Yuan, Yangyang Lu, Chenze Zhu, Chen Zhang, Haifeng Lue, Xiangnan Zhang

**Affiliations:** ^1^ Institute of Pharmacology & Toxicology, College of Pharmaceutical Sciences Zhejiang University Hangzhou China; ^2^ School of Pharmacy Hangzhou Medical College Hangzhou China; ^3^ Jinhua Institute of Zhejiang University Jinhua China

**Keywords:** anti‐manic drugs, autophagy, circadian rhythm, mania

## Abstract

**Aims:**

Mania is a prevalent psychiatric disorder with undefined pathological mechanism. Here, we reviewed current knowledge indicating the potential involvement of autophagy dysregulation in mania and further discussed whether targeting autophagy could be a promising strategy for mania therapy.

**Discussions:**

Accumulating evidence indicated the involvement of autophagy in the pathology of mania. One of the most well‐accepted mechanisms underlying mania, circadian dysregulation, showed mutual interaction with autophagy dysfunction. In addition, several first‐line drugs for mania therapy were found to regulate neuronal autophagy. Besides, deficiencies in mitochondrial quality control, neurotransmission, and ion channel, which showed causal links to mania, were intimately associated with autophagy dysfunction.

**Conclusions:**

Although more efforts should be made to either identify the key pathology of mania, the current evidence supported that autophagy dysregulation may act as a possible mechanism involved in the onset of mania‐like symptoms. It is therefore a potential strategy to treat manic disorder by correting autophagy.

## Graphical abstract

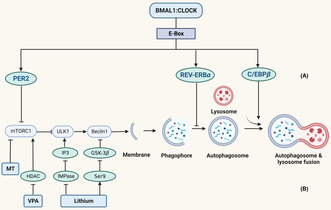

## 1 INTRODUCTION

Bipolar disorder (BD) is a mental disorder characterized by periods of depression and periods of mania that each lasts from days to weeks.^1^ It is generally accepted that mania is inevitably accompanied by depression episodes in patients, known as BD. Notably, most of the animal models only exhibit manic or depressive symptoms, and the animal models reflecting the phenotypes of BD are still lacking.^2^ Mania is featured by abnormally elevated arousal, affect, and energy level.^3^ The symptoms of mania include elevated mood, increased energy, hyperactivity, and decreased desire for sleep. However, the mechanisms underlying mania have not been fully understood. In patients, mania is highly consistent with dysfunction in several brain regions, including the prefrontal cortex, basotemporal cortex, and basal ganglia.^4^ At the cellular level, aberrant neurotransmitter functioning in the central nervous system (CNS), including dopamine, glutamate, and gamma‐aminobutyric acid, is closely related with mania.^5^ In terms of genetic level, CLOCK gene deletion in mice is linked to mania‐like behavioral changes and can be reversed by lithium treatment; the knockout of metabotropic glutamate receptor 6 (mGluR6), pituitary adenylate cyclase‐activating peptide ameliorate mania‐like behaviors in mice.^6^ Nevertheless, it remains elusive how the dysregulation of these brain regions, neurotransmitters, and genes results in mania. The therapeutic drugs for mania may provide molecular insight into the pathological mechanisms of mania. For instance, DA transporters increased (60–90%) in all sections of the rostral and caudal neostriatum with chronic lithium treatment (28 days).^7^ Enhanced phosphatidylinositol signaling reduced the efficacy of carbamazepine and valproate.^8^ However, these drugs show drawbacks, such as the narrow therapeutic window of lithium salts, the toxicity of valproate on the liver and the kidney, etc. Insight into the mechanics of mania is crucial to reduce side effects. Therefore, in order to develop safer and more effective therapeutic drugs, more efforts have to be paid to understand the pathological mechanisms of mania.

Autophagy refers to an intracellular process that removes unnecessary or dysfunctional components through a lysosome‐dependent manner. The molecular details of autophagy have been summarized elsewhere.^9^ Given the extensive studies of autophagy in neurodegenerations, recent evidence implied dysregulation of autophagy in affective disorders, including mania. Both the protein and mRNA levels of LC3 and p62 decreased in peripheral blood mononuclear cells from patients with BD. In addition, the genes involved in autophagy activation were found downregulated in blood samples from patients with BD. These studies implied a potential link of autophagy inhibition with mania.^10^, ^11^ Indeed, autophagy enhancers reduced mania‐like aggression and reward‐seeking behavior in mice.^12^ Conversely, dysregulation of autophagy seems to underlie the development of mania. For instance, circadian rhythm disruption, either considered as a causing factor or a consequence of mania‐like symptoms, has been found to regulate autophagy initiation, maturation, and autolysosome formation through different signaling pathways.^13^, ^14^, ^15^, ^16^ Moreover, studies have shown that anti‐manic drugs, such as lithium, carbamazepine, valproate, and melatonin, may activate autophagy, which have been proposed to contribute to their efficacy for mania treatment.^17^, ^18^, ^19^, ^20^ In addition, autophagy dysregulation leads to abnormalities in mitochondrial quality control, neurotransmission, and ion channel function, which may dedicate to the development of mania.^21^, ^22^, ^23^ These lines of observation raised a hypothesis that autophagy dysregulation contributes to the pathology of mania disorder, although the underlying mechanisms remains largely unclear. Here, we review the present evidence for the potential involvement of autophagy in mania and further discuss whether and how autophagy regulation may contribute to the anti‐manic drugs development.

## 2 RHYTHMIC REGULATION OF AUTOPHAGY IS DISRUPTED IN MANIA

The circadian rhythm controls the biological clock and a range of cellular activities.^24^ Since the discovery of the Period gene, many clock genes encoding transcription factors (TFs) that compose the molecular clock have been cloned and characterized, among which the most important is BMAL1:CLOCK transcription complex.^25^ It binds to the E‐box, which activates the transcription of downstream genes such as CRY and PER.^26^ The above process mainly occurs during the daytime, and the proteins formed by those genes are generally degraded in the cytoplasm by the proteasome. On the contrary, when it comes to late afternoon, PER and CRY form heterodimerization to prevent their degradation. This heterodimerization translocates to the nucleus and inhibits the activity of the BMAL1:CLOCK complex by inducing the transcription of REV–ERBα and REV–ERBβ, thereby affecting mammalian circadian rhythms.^27^, ^28^

Circadian rhythm dysregulation is thought to be either a precipitating factor or a consequence of mania‐like symptoms.^29^, ^30^, ^31^ In animals, mice carrying a deletion at exon 19 of the circadian locomotor output cycles Kaput gene (Clock^delta19^) were observed to exhibit mania‐like behaviors and recognized as a model for BD.^32^ In manic patients, the abnormality of circadian rhythms usually are well characterized by insomnia,^33^ disturbed sleep–wake rhythms^34^ and increased anxiety about sleep.^35^ The possible involvement of circadian rhythm dysregulation in mania includes the following two ways. At the molecular level, melatonin is secreted abnormally in patients with BD. Also, melatonin secretion levels significantly increased and peaked prior to the control in mania states.^34^ In furthermore, it has been shown that genes regulating biological rhythms, such as CLOCK, PER3, and ARNTL, are involved in the prophylactic effects of lithium^35^ (the prophylactic effects of lithium refer to the phenomenon that using maintenance doses of lithium are effective in preventing the recurrence of manic episodes in patients).^36^ However, the accurate mechanism of disrupted circadian rhythm underlying mania still remains elusive.

**FIGURE 1:**
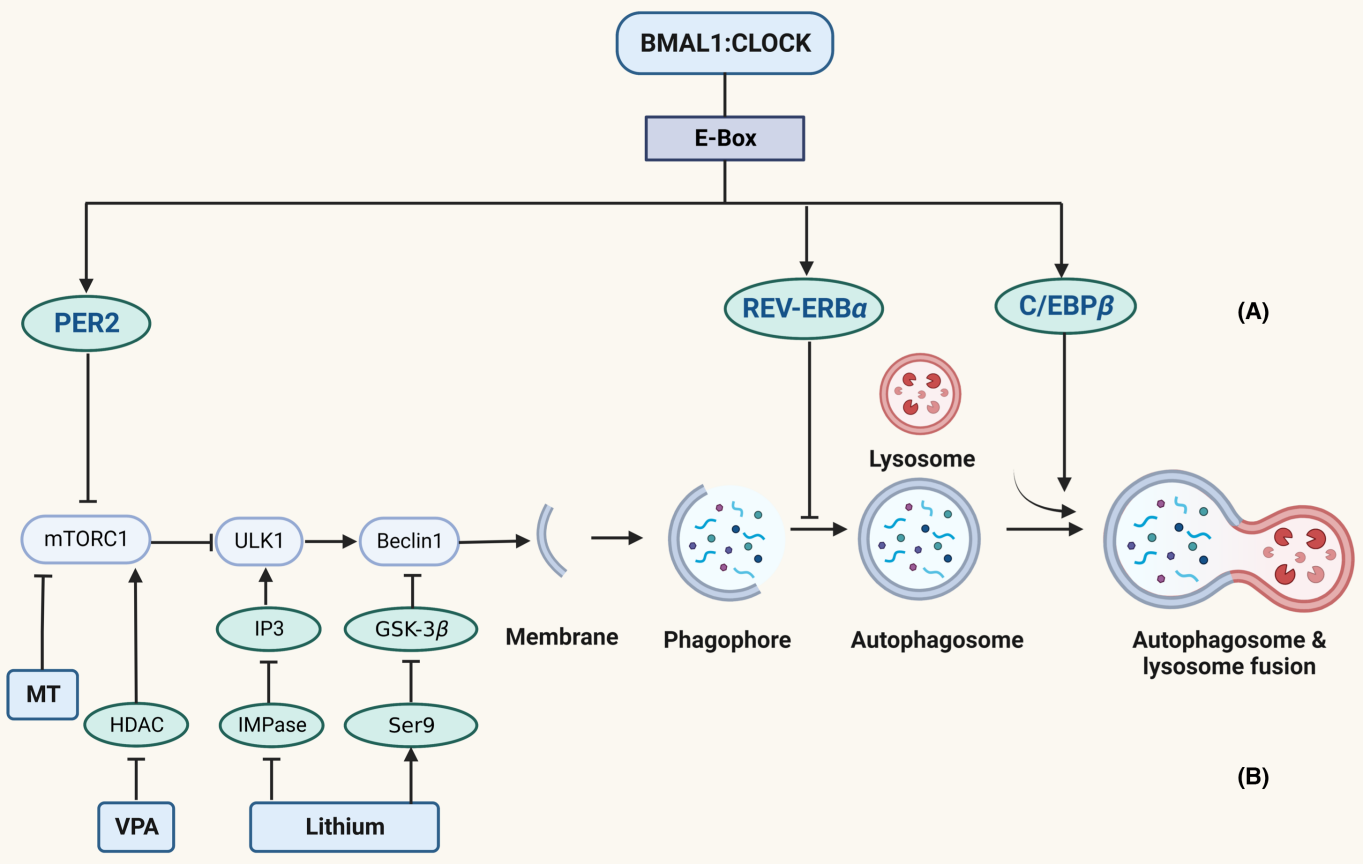
Autophagy is regulated by circadian rhythm and anti‐manic drugs. (A) Circadian rhythms can regulate the occurrence of autophagy in three stages: PER2 can regulate the activation of autophagy by inhibiting mTOR; REV–VERBα can regulate the maturation of autophagosome through binding to TFEB or TFE3 (TFEB/TFE3); C/EBPβ can regulate the formation of autolysosome. (B) A variety of evidence coming from pharmacological studies supports the involvement of autophagy in mania, including lithium, carbamazepine, valproate, and melatonin. These drugs all upregulate autophagy through multiple mechanisms: Lithium inhibits GSK3β and IMPase; valproate (VPA) inhibits histone deacetylase (HDAC) to inhibit mTOR; melatonin (MT) downregulates mTOR.

Emerging evidence revealed that autophagy dysregulation may serve as an underlying mechanism by which abnormal circadian rhythms trigger mania. It is widely known that autophagy is rhythmically regulated. The volume and quantity of autophagic vacuoles changed with light–dark, day and night in rat's heart.^15^ Using more specific molecular markers, autophagic flux was evaluated by the degradation rate of LC3‐II, and it was found that autophagic flux peaked in the afternoon and decreased to a lower extension during the dark phase in several mouse tissues, including the liver, heart, and skeletal muscle.^37^ Circadian rhythms can regulate autophagy mainly in three stages: the activation of autophagy,^13^ the maturation of phagophore,^14^ and the formation of autophagosome^16^ (Figure 1A). In each stage, circadian rhythm regulates autophagy through different mechanisms, supporting the hypothesis that autophagy may be one of the mechanisms by which circadian rhythms cause mania‐like symptoms.

### 2.1 Initiation stage

In the initiation stage, the circadian clock gene PER2 inhibited mTORC1 and thus promoted autophagy.^13^ PER2 is a core clock protein and its expression can promote autophagy. Transient overexpression of PER2 significantly increased autophagic flux.^38^ In HeLa cells, the effect of PER2 on LC3‐II and autophagosome adaptor p62 abundance were examined. It was found that ectopic expression of PER2 increased LC3‐II but decreased p62 protein, suggesting autophagy activation during the initial stage of circadian rhythms. Moreover, the number of autophagosomes dramatically reduced in the situation of PER2 deficiency.^13^ mTOR pathway activated autophagy by sensing nutrient limitation.^39^ mTORC1 consists of mTOR, RAPTOR, PRAS40, DEPTOR, and mLST8 (also known as GL). mTORC1 integrates intra‐ and extracellular signaling to suppress autophagy by phosphorylating ULK1 and thus serves as a key factor in autophagy regulation.^40^

It is shown that PER2 interacted with mTORC1 and initiated autophagy. The results of co‐immunoprecipitation assays showed that strong interaction of PER2 with RAPTOR (a component of mTOC1) and thus regulated mTORC1 activity. Moreover, it is also found that immunoprecipitated Myc–mTOR proteins retrieve a large amount of PER2 proteins instead of other clock genes such as CLOCK and CRY, indicating the particular interaction between PER2 and mTOC1. Actually, PER2 acts as a scaffold protein, binding Tsc1, Raptor, and mTOR together to suppress the activity of the mTORC1 complex specifically. Overexpression of PER2 was observed to reduce the phosphorylation of mTOR and S6k significantly, suggesting activated mTORC1 and enhanced Tsc1–mTOR association. However, the interaction between endogenous Tsc1 and mTOR proteins in MEFs is completely disrupted by PER2 depletion, and PER2 inhibition of mTORC1 activity is completely abolished in Tsc1‐deficient MEFs.^13^ Taken together, these evidences revealed the positive control of autophagy initiation by PER2 via inhibiting mTORC1 activity.

### 2.2 Maturation of autophagosome

Besides PER2, in the stage of autophagosome maturation, the circadian clock component REV–ERBα bond to TFE and thus regulate autophagy.^14^ REV–ERBα upregulated the expression of circadian rhythm‐related genes as a transcriptional factor.^41^ It showed that the expression of REV–ERBα protein in lungs has an obvious circadian rhythm and the rhythmic diurnal variation disappears then REV–ERBα is knocked out.^42^ It is found that TFEB or TFE3 (TFEB/TFE3) binding with REV–ERBα are the key regulatory factors for autophagy.^43^ TFEs exhibited circadian activation throughout the 24‐h cycle and were responsible for the rhythmic induction of autophagy genes during the light phase. The level of TFE3 was directly correlated with the level of LC3‐II and p62, and autophagy was suppressed with TFE depletion.^14^ TFEs control autophagosome maturation, including the regulation of membrane recruitment of key components during fusion events, as well as transcriptional regulation.^44^

It is also found that REV–ERBα plays a crucial role in the regulation of specific autophagy genes, including vesicle nucleation and expansion, autophagosome formation, etc., which suggested the connection between REV–ERBα and autophagy.^15^, ^45^ REV–ERBα can regulate autophagy via binding with TFEB/TFE3. Site‐binding of TFE3 and REV–ERBα is observed in promoter regions of genes involved in circadian rhythm, autophagy, and lysosomal biogenesis (e.g., LAMP1, Mcoln1, Vps33a, ATG3, ATG5, CTSL, and Gabarapl1). Meanwhile, ChIP analysis shows several ChIP‐seq peaks of TFEB/TFE3 in the REV–ERBα promoter, which demonstrated direct regulation of REV–ERBα by TFEB/TFE3. Furthermore, the luciferase assay confirmed that TFEB/TFE3 directly activated the REV–ERB promoter which showed a dynamic balance between TFEB/TFE3 and REV–ERBα, and this process might be responsible for the oscillation of autophagy activation.^14^ Of note, the silence of REV–ERBα also reinforces the expression suggesting the REV–ERBα participates in the process of the regulation of autophagy. In Hepa1‐6 cells, reduced level of REV–ERBα mRNA was found with overexpression of TFEB or TFE3.^14^ In sum, REV–ERBα promoted autophagy through binding to basic helix–loop–helix (bHLH) MiT–TFE TFs TFEB and TFE3.

### 2.3 Formation of autolysosome

The formation of autolysosomes can also be regulated by CCAAT enhancer‐binding protein (C/EBPβ), which is a TF known as a mammalian clock regulator. C/EBPβ also participates in the process of apoptosis and autophagy, coordinating the rhythmic expression of autophagic genes and integrating circadian signals into autophagy.^46^ It showed that C/EBPβ protein expression had a cyclic rhythm and reached the maximum in the dark phase. C/EBPβ is located downstream of circadian and nutritional signaling pathways.^47^ Compared with the control group, adenoviral‐mediated expression of C/EBPβ significantly increased LC3‐II levels in transduced hepatocytes, providing direct evidence for the involvement of C/EBPβ in the circadian regulation of autophagy. In addition, mice lacking functional liver clocks were characterized with altered C/EBPβ levels, disrupted autophagy rhythmic regulation, and decreased autophagy gene expression.^15^

C/EBPβ may regulate autophagy via serval pathways. First, C/EBPβ stimulated the transcription of autophagic genes by directly binding with promoters of autophagy genes, including Gabarapl1, Bnip3, and Ctsl. These genes are components in the process of autophagy or mitophagy, and their transcription was activated by C/EBPβ. By constructing a luciferase reporter, it is shown that C/EBPβ significantly induced the expression of these genes.^15^ C/EBPβ served as a key effector in autophagy by promoting the fusion of lysosomes with autophagosomes to form autolysosomes. Using plasmid ptfLC3 expressing a mRFP‐GFP‐LC3 fusion protein, autophagosome‐lysosome fusion in PC3 was evaluated. Compared with control cells, deletion of C/EBPβ caused a 4‐fold rise in the proportion of RFP/GFP‐positive autophagosomes and a reduction of RFP‐positive autolysosomes. All these impairments indicate that C/EBPβ is critical for basal autophagy by regulating autophagosome–lysosome fusion.^48^

However, as mentioned above, the exact mechanism of autophagy is directly related to specific brain region, but almost all evidence happens in the peripheral surroundings instead of the brain. Thus, direct evidence in the brain region is still lacking. Moreover, according to current research results, it is undeniable that there is still a lack of valid direct evidence for the involvement of circadian rhythms in the pathogenesis of mania‐like symptoms, since not all mice with knockout of circadian genes show mania‐like symptoms. CLOCK, a key gene involved in circadian rhythms, has been experimentally demonstrated that after its expression was inhibited by the introduction of pClock.shRNA pelleting, the suppressor mice showed little change in activity compared with mice with the introduction of pCtrl.shRNA control plasmid.^49^ The PER3 knockout mice had a slightly shorter free‐running period than normal mice and showed a mild phenotype without mania‐like symptoms.^50^ Moreover, the prolonged non‐eye movement sleep time in CRY1 and CRY2 double knockout mice involved in circadian rhythm was not compatible with the reduced non‐eye movement sleep time of mania‐like symptoms.^51^

## 3 THERAPEUTIC DRUGS FOR MANIA REGULATING AUTOPHAGY

There is a variety of evidence coming from the pharmacological studies supporting the involvement of autophagy in mania, including lithium, carbamazepine, valproate, and melatonin (Figure 1B). These drugs all upregulate autophagy through multiple mechanisms (Table 1). Lithium inhibits GSK3β and IMPase. Both carbamazepine and valproate decrease inositol phosphate levels, and valproate also inhibits histone deacetylase. Besides, melatonin downregulates mTOR and upregulates PI3K levels. All these evidences suggest that autophagy may account for their manic therapeutic functions.

**TABLE 1:** Summary of anti‐manic drugs with autophagic effects.

Drug	Target	Mechanisms	Regulation of autophagy
Lithium(Li)	GSK3β	GSK3β inhibition leads to elevated Bif‐1 and interacts with the Beclin‐1‐VPS complex that induces autophagy. Li enhances phosphorylation at the Ser^9^ residue and thus inhibits GSK3β activity	Activation
	IMPase	IMPase catalyzes the hydrolysis of inositol monophosphate (IP1) to free inositol required for the phosphatidylinositol signaling pathway to induce autophagy. Li leads to free inositol depletion and thus downgrades inositol and IP3 levels	Activation
Carbamazepine(CBZ)	IP3	CBZ activates AMPK by decreasing inositol and IP3 levels. mTORC1 phosphorylation is inhibited by AMPK after AMPK is activated, and ULK1 can then interact with and be phosphorylated by AMPK. Activated ULK1 initiates autophagy	Activation
Valproate(VPA)	IP3	As above	Activation
	HDAC	VPA is a histone deacetylase (HDAC) inhibitor. HDAC inhibitor increases histone acetylation, which disrupts the binding of histones to nuclear DNA. This exacerbates DNA susceptibility to transcription and may inhibit mTORC1 signaling to enhance autophagy. HDAC5 regulates mTORC1 signaling by regulating the phosphorylation of Ser^792^. VPA inhibits HDAC5 activity, leading to Lys840 and increased acetylation of Ser^792^, which leads to inhibition of mTORC1 signaling	Activation
Melatonin(MT)	PI3K/AKT/mTOR	MT downregulates the PI3K/AKT/mTOR pathway	Activation

Lithium has been clinically used as the preferred drug of choice for the treatment of BD for more than half a century, but the mechanism of lithium remains unclear. Since studies have reported that lithium induces autophagy and enhances the degradation of mutant Huntingtin via mTOR‐independent pathways in non‐neuronal and neural precursor cells,^17^ a growing amount of literature has demonstrated the autophagy‐regulating role of lithium. It was shown that lithium regulates autophagy through two pathways: inhibition of GSK3β and inositol monophosphatase (IMPase). GSK3β activity is modulated by phosphorylation of amino acid residues at the locus: phosphorylation of Ser^9^ residues downregulates GSK3β; conversely, phosphorylation of Tyr^216^ residues upregulates GSK3β. It was shown that lithium enhances autophagy by increasing phosphorylation at the Ser^9^ residues to inhibit GSK3β.^52^, ^53^ GSK3β inhibition leads to elevated Bif‐1 and interacts with the Beclin‐1‐VPS complex thus induces autophagy.^54^ On the other hand, IMPase catalyzes the hydrolysis of inositol monophosphate (IP1) into free inositol. Inositol produces myo‐inositol‐1,4,5‐trisphosphate (IP3) through the phosphatidylinositol signaling pathway.^55^ Treatment with myo‐inositol or PEI alone, which elevated IP3 levels, inhibited autophagy.^17^ Moreover, increasing inositol or IP3 levels reversed the autophagic effect of lithium.^17^ Thus, lithium may enhance autophagy through inhibiting IMPase to deplete free inositol and reduce IP3 levels. The above two pieces of evidence demonstrate that lithium upregulates autophagy through phosphatidylinositol signaling pathway.

Carbamazepine, an antiepileptic drug, is also one of the drugs commonly used to treat manic symptoms in BD. It was shown that a significant increase in the number of autophagosomes and autophagic lysosomes was observed in cells treated with Carbamazepine (50 μM, 6 h) and in positive control rapamycin‐treated (100 nM, 2 h) cells, indicating that Carbamazepine enhances autophagy.^18^ In the animal model, mice treated with 50 mg/kg/day of Carbamazepine for 4 weeks showed an upregulation of LC3II levels compared to controls.^18^ Similar to lithium, Carbamazepine induces autophagy through phosphatidylinositol signaling pathway.^56^, ^57^, ^58^, ^59^ Carbamazepine can activate AMPK by decreasing inositol and IP3 levels. mTORC1 phosphorylation is inhibited by AMPK after AMPK is activated, and ULK1 can then interact with and be phosphorylated by AMPK. Activated ULK1 initiates autophagy and ultimately upregulates autophagy. However, the increase in autophagic flux observed in carbamazepine‐treated cells disappeared after excess inositol treatment,^18^ indicating that excess inositol blocked the ability of carbamazepine to enhance autophagy. In addition, it has been shown that the stimulatory effect of carbamazepine on autophagy is dose‐dependent and further enhances the already upregulated autophagy in patients.^60^

Valproate is an antiepileptic drug that is also often used in combination with lithium for mania, but its mechanism remains unclear. In male C57BL/6 mice, it has been shown that valproate treatment (20 μg/mL) increased LC3II expression.^19^ LC3II expression was significantly higher in the lithium (10 μg/mL) and valproate (20 μg/mL) combination treatment group than in controls while the use of autophagy inhibitors wortmannin decreased the increase in LC3II levels.^19^ Similarly, valproate upregulates autophagy via the phosphatidylinositol pathway.^61^ In addition, valproate is a broad‐spectrum histone deacetylase inhibitor (HDACI). HDAC5 modulates the phosphorylation of Ser^792^ to regulate mTORC1.^62^ mTORC1 and reduces the activity of ULK1 by phosphorylating ULK1 and Atg13 thus inhibits autophagy.^63^ Inhibition of HDAC5 by valproate results in increased phosphorylation of Ser^792^, which upregulates autophagy by mTORC1.

Patients with BD exhibit not only circadian rhythm disruption but also melatonin downregulation.^20^ As a circadian rhythm regulator, melatonin is also commonly used as an adjunctive therapy for mania. Melatonin may upregulate autophagy by the phosphatidylinositol 3‐kinase (PI3K)/protein kinase B (AKT)/mTOR signaling pathway.^64^, ^65^ It was found that treatment with melatonin (12.5 mg/kg/day) by intraperitoneal injection (i.p.) for 7 days significantly increased the level of LC3II in cells compared to the controls. Conversely, the expression of p‐PI3K, p‐mTOR, and p‐AKT was reduced in the melatonin group.^66^ PI3K/AKT/mTOR serves as a negative modulator of autophagy. Thus, melatonin may promote autophagy by blocking PI3K/AKT/mTOR pathway. However, combining a melatonin selective receptor antagonist luzindole (5 μM, 10 μM, and 15 μM, 30 min) with melatonin (200 μM, 12 h) did not reduce the melatonin‐induced increase in LC3II levels.^67^ Thus, melatonin activation of autophagy may not be receptor mediated.

Overall, most of the therapeutic drugs for mania showed potential effects on autophagy upregulation. Nevertheless, there is no direct evidence to demonstrate the necessity of autophagy in the pharmacodynamics of these drugs. This may be a coincidental phenomenon and does not exclude that they may have other pharmacological activity. In addition, atypical antipsychotics are clinically used to treat mania. They have also been found to have a modulatory effect on autophagy. For example, olanzapine increased LC3II level and decreased p62 level of the glioma cell lines.^68^ However, although various anti‐psychotics can modulate autophagy,^69^ not all of them can treat mania. Atypical anti‐psychotics may exert their anti‐manic effects through dopaminergic and 5‐HT, etc.^70^, ^71^ Therefore, autophagy may not play a dominant role in the anti‐manic efficacy of the above drugs, and further studies on anti‐manic drugs modulating autophagy are supposed to focus on the role of autophagy in mania.

## 4 POSSIBLE PATHOLOGICAL MECHANISMS BY WHICH AUTOPHAGY AFFECTS MANIA

Abnormalities in mitochondrial quality control, neurotransmission, and ion channel function are associated with mania. Evidence highlights that autophagy may play a role: Mitochondrial autophagy is disrupted in patients with BD; dysregulation of autophagy causes neurotransmitter release from synaptic vesicles; cytoplasmic K^+^ and Ca^2+^ dysregulation is related to autophagy. These further suggest that autophagy may involve mania in various ways.

### 4.1 Mitophagy deficiency underlying mania

Mitochondria is a crucial organelle for neuronal survival and function. Recent evidence indicated an intimate association of mitochondrial dysfunction with psychiatric disorders, including mania. Patients with BD have disrupted mitochondrial morphology, content, and distribution in the prefrontal cortex compared to healthy individuals.^72^, ^73^ In addition, mitochondrial metabolism and ATP levels increased in the manic phase of patients with BD.^74^ Besides, their mitochondrial DNA copy number is significantly lower, and the DNA damage positively correlates with the extent of BD.^75^ These studies implied that compromised mitochondrial quality underlies the pathogenesis of mania.

In neurons, mitochondrial quality control is executed by degrading damaged or superfluous mitochondrial via autophagy‐dependent manner, termed mitophagy.^76^ Mitophagy dysfunction is closely related with various neurological disorders. Emerging evidence implied the involvement of mitophagy dysfunction in affective disorders as well.^21^ Abnormalities in mitochondrial autophagy can lead to abnormalities in quality control by preventing the removal of damaged parts of mitochondria.^76^ All of the above suggests that abnormalities in mitochondrial quality control may be related to autophagy.^77^

Ubiquitination of the mitochondrial outer membrane proteins by PINK1/Parkin pathway serves as a trigger to induce mitophagy. PINK1 recruits Parkin to mitochondria, where Parkin further ubiquitinates its substrates as an E3 ligase. Once mitochondria are ubiquitinated, they can bind to the autophagy receptor and LC3, thereby driving their engulfment by autophagosomes.^51^ PINK1 or Parkin loss‐of‐function mutation is high risk factor for PD, which is not only characterized by paralysis but also psychiatric disorders including memory loss, depression, pain, and sleep disturbances.^78^ Nevertheless, the direct link between PINK1/Parkin dysfunction and BD has not been established. Intriguingly, in a one‐year prospective study, 18 (16.2%) out of 111 consecutive patients with PD developed mania.^79^ Moreover, in another study, 14 of 17 patients (82%) developed hypomania/mania after the bilateral deep brain stimulation (DBS) of the subthalamic nucleus (STN) treatment,^80^ implying a potential PINK1/Parkin‐related mitophagy dysregulation in mania onset.

As mentioned above, abnormal autophagy leads to abnormal mitochondrial mass, thus serving as one of the possible mechanisms for its triggering of mania.

### 4.2 Autophagy regulation of neurotransmission

As mentioned above, mania may be related to abnormalities in the function of neurotransmitters in the CNS such as dopamine, glutamate, and gamma‐aminobutyric acid. For instance, it has been found that enhanced dopamine neurotransmission can lead to mania‐like behavior in rodents.^81^

The release of presynaptic neurotransmitters is fine‐tuned through the secretory cycle of synaptic vesicles. This requires the dynamic interplay of multiple mechanisms of secretory trafficking pathway and autophagy. In line with this, dysregulation of autophagy quite stably impaired the secretory and trafficking pathways, which eventually precipitates synaptic dysfunction.^22^ Indeed, several organelles of the secretory pathway work together to provide a membrane source for autophagic vesicles and synaptic vesicles.^82^ The overlap of membrane sources of autophagic vesicles and synaptic vesicles may be regulated by similar molecular complexes. For instance, the UNC51‐like kinase 1/2 complex promotes the initiation of autophagy.^83^ Meanwhile, loss of UNC51 inhibits axonal transport of vesicles in addition to impairing autophagy initiation. In mice lacking ATG7, specifically within dopaminergic neurons (Atg7‐DAT‐Cre mice), the amplitude of the dopaminergic signal evoked by electrical stimulation was 54% greater than in DAT‐Cre mice. The enhancement of autophagy with rapamycin decreases the number of dopamine synaptic vesicles 25%, and dopamine release decreased by about 25% correspondingly.^84^

Thus, autophagy dysregulation may lead to mania through abnormal transmission of dopamine and other neurotransmitters.

### 4.3 Autophagy regulates mania‐associated ion channels

Studies have shown that primary or secondary dysfunction of Na, K‐ATPase plays a predisposing or more direct etiological role in BD. Specifically, Ouabain (OUA), an inhibitor of Na+/K+ ‐ATPase enzyme, induced mania‐like behavior in mice, which was reversed by lithium.^85^ K+ deprivation has been found to be a potent inducer of autophagy. Mutations in the endolysosomal K+ ‐selective channel KEL impair autophagosomal–lysosomal fusion.^23^

It has also been found that intracellular calcium signaling is elevated in patients with mania.^86^ Also, elevated cytoplasmic Ca^2+^ triggers autophagy. Calcium‐induced activation of autophagy is dependent on the core Atg protein and CaMKKβ signaling pathways, and elevation of Ca^2+^ mobilizers TG and ATP can deduce inhibition of Atg7 and CaMKKβ.^87^ In addition, increased cytoplasmic Ca^2+^ may also induce autophagy by inhibiting the activity of mTOR^88^ and AMPK.^89^, ^90^ Not only that, endoplasmic reticulum‐localized Bcl‐2 can interfere with endoplasmic reticulum calcium mobilization and thus inhibit autophagy. This mechanism relies on the regulation of AMPK activity by CaMKKβ, which in turn inactivates mTORC1 to inhibit cell growth and promote autophagy.

Therefore, autophagy dysfunction may trigger abnormal ion channel function, which may be one of the causes for mania.

## 5 CONCLUSION

As mentioned above, we have listed the evidence that autophagy may be involved in mania through different perspectives. Circadian rhythms may have an impact on mania by controlling various stages of autophagy. In addition, drugs used to treat mania can upregulate autophagy, suggesting that they may exert their therapeutic effects through autophagy. Dysregulation of autophagy leads to abnormalities in mitochondrial quality control, neurotransmission, and ion channel function, which may thereby affect manic progression. Although, we still lack direct evidence for the conjecture that mania is involved in autophagic regulation. For example, it was found that mice do not exhibit mania after knocking out autophagy‐related genes (e.g., ATG5, ATG7). One possible explanation can be that different brain regions and loops which are responding to mania have diverse and specific autophagic changes instead of a brain‐wide and universal regulation of autophagy. Hypothalamic–pituitary‐adrenergic (HPA) axis dysfunction and the suprachiasmatic nucleus (SCN) in the anterior hypothalamus are associated with BD, and the latter has been firmly established as the primary circadian pacemaker in mammals. Follow‐up studies may provide new evidence for the association between autophagy and mania by intervening in key brain regions (e.g., hypothalamus) and loops associated with mania. What's more, current pathological mechanisms surrounding autophagy are mainly focused on neurodegenerative diseases, the evidence for the involvement of autophagy in the pathology of mania is far from fully understood, and lack of consensus.

Overall, further research is still needed to demonstrate the pathological mechanisms of autophagy involved in the development of mania‐like symptoms. However, there is no drugs available for the treatment of mania by modulating autophagy. Given the potential efficacy of autophagy regulators in treating CNS‐related diseases, it is plausible to find promising therapeutic drugs for mania from these autophagy regulators. In summary, although vigorous attempts need to be made to either verify or falsify key pathology, current evidence supporting autophagy as a possible mechanism involved in the onset of mania‐like symptoms. It could be a promising strategy to treat BD by autophagy regulators.

## CONFLICT OF INTEREST STATEMENT

We declare that we do not have any commercial or associative interest that represents a conflict of interest in connection with the work submitted.

## Data Availability

Data sharing not applicable to this article as no datasets were generated or analysed during the current study.
